# Comparison of ocular biometrics between diabetic and non-diabetic adults: a population-based study

**DOI:** 10.1038/s41598-026-56429-1

**Published:** 2026-06-08

**Authors:** Elham Angouraj Taghavi, Mohammad Hassan Emamian, Hassan Hashemi, Akbar Fotouhi

**Affiliations:** 1 Student of Research Committee, School of Medicine, Shahroud University of Medical Sciences, Shahroud, Iran; 2 Ophthalmic Epidemiology Research Center, Shahroud University of Medical Sciences, Shahroud, Iran; 3 Noor Ophthalmology Research Center, Noor Eye Hospital, Tehran, Iran; 4 Department of Epidemiology and Biostatistics, School of Public Health, Tehran University of Medical Sciences, Tehran, Iran

**Keywords:** Biometry, Diabetes, Axial length, Iran, Population-based study, Diseases, Endocrinology, Health care, Medical research

## Abstract

This study aimed to compare ocular biometrics between diabetic and non-diabetic individuals. Understanding these biometric differences may inform cataract surgery planning and glaucoma risk assessment in diabetic patients. Participants were drawn from the second phase of the Shahroud Eye Cohort Study. Diabetes mellitus was diagnosed based on fasting blood sugar (FBS) levels of ≥ 6.99 mmol/L, HbA1c levels of ≥ 6.5%, or the use of hypoglycemic medications. Ocular biometrics were recorded using a Lenstar optical biometer. Differences in ocular biometrics between participants with and without diabetes were analyzed using t-tests and linear regression models. Among the 4452 participants, aged 45–69 years. Axial Length (AL) (Mean Difference (MD) = − 0.09 mm, 95% CI − 0.16 to -0.03), Corneal Diameter (CD) (MD = − 0.10 mm, 95% CI − 0.13 to − 0.07), Aqueous depth (AD) (MD = − 0.03 mm, 95% CI − 0.06 to − 0.01), were higher in non-diabetics, while central corneal thickness (CCT) (MD = 5.90 μm, 95% CI 3.70 to 8.10), and lens thickness (LT) (MD = 0.06 mm, 95% CI 0.03 to 0.08) were thicker in diabetics. In the multiple linear regression model, AL, AD, and CD were negatively associated with diabetes, while LT and CCT was positively associated with diabetes. In conclusion, among ocular biometrics, diabetic individuals had thicker lens and central corneas, while AL, AD, and CD were lower. Other ocular biometrics did not differ significantly in diabetics than non-diabetics. While the observed differences are statistically significant, their clinical relevance is minimal due to small effect sizes.

## Introduction

 Globally, almost 830 million individuals are living with diabetes in 2022 and the disease prevalence is rapidly increasing especially in middle- and low-income countries^1^. Diabetes mellitus can lead to ocular complications such as diabetic retinopathy, cataracts, glaucoma, and ocular surface diseases^2^. Despite the progressive nature of these ocular complications, they are preventable and treatable in the early stages^2^. Global advances in ophthalmology have led to an extensive need to determine the ocular biometric components. These biometrics are used in diagnostic and clinical fields of ophthalmology, such as retinal detachment risk calculation^3^, staphyloma^4^, lens power calculation to determine refractive power, and for minimizing surgery-induced astigmatism^5,6^. A previous study noted a shorter axial length (AL) and shallower anterior chamber depth (ACD) in type 2 diabetic patients when compared to the healthy population^7^. Another study found a potential protective of longer AL and deeper ACD against diabetic retinopathy progression^8^. There is association between lens power (LP) and axial length-to-corneal radius ratio (AL/CR ) in diabetic retinopathy^9^. Diabetes is also associated with increased central corneal thickness (CCT)^10^. However, the drawbacks of studies in this field are non-standard measurements, small sample sizes, and controversial results^9,11,12^. This study compared and reported biometric components in both diabetic and non-diabetic individuals with standard methods and appropriate sample size. Identifying diabetes-associated ocular biometric changes could help optimize intraocular lens power calculation and improve monitoring of diabetic eye disease.

## Methods

The present study was based on the data from the second phase of the Shahroud Eye Cohort Study which was conducted in 45-69-year-old population of Shahroud, Iran. The second phase took place approximately five years after baseline. The cohort details have been published elsewhere^13^. In brief, in the first (baseline) phase of the study, participants were enrolled using the random cluster sampling method. Individuals were selected from different areas of Shahroud, northeast Iran in 300 clusters. At least 20 individuals between the 40–64 years old were randomly selected from each cluster. A total of 6311 people were invited for optometric and ophthalmic examinations and 5190 (82% response rate) accepted to participate in the first phase of the study in 2009. All participants in the first phase were invited for the second phase 5 years later in 2014, and 4737 (91.3% response rate) of them accepted the invitation. Participants underwent optometric and ophthalmic examinations. Blood samples were collected prior to ophthalmic examination. Participants fasted for 8 to 12 h before blood draw, verified by self-report. All collections performed between 8:00 and 10:00 AM). Fasting blood sugar (FBS), lipid profile, and HbA1c were measured from the same blood sample. Diabetes mellitus was defined as FBS ≥ 6.99 mmol/L, HbA1c ≥ 6.5%, or self-reported use of glucose-lowering medications. Individuals with a systolic blood pressure ≥ 140 mmHg or diastolic blood pressure ≥ 90 mmHg, or those taking antihypertensive medications, were classified as having hypertension.

Ocular biometric components were measured using the Lenstar LS 900 optical biometer (Allegro BioGraph, WaveLight AG, Erlangen, Germany). This device uses optical low-coherence reflectometry (OLCR) to obtain simultaneous measurements of ocular biometrics. All measurements were performed by a trained nurse following a standardized protocol. Participants were seated comfortably with their chin and forehead positioned on the device’s rest and were instructed to fixate on the internal target. The device was set to acquire three measurements with acceptable quality per eye per parameter. Acceptable quality was defined as a device-reported quality. If three acceptable measurements could not be obtained, the eye was excluded from analysis. The device was calibrated daily before starting measurements. The mean of the three measurements was used for analysis. Participants with prior ocular surgery (including cataract, refractive, glaucoma, or vitreoretinal surgery), corneal disease (keratoconus, scar, edema, dystrophy), contact lens wear within 3 months, were excluded from analysis. These exclusion criteria were applied to both diabetic and non-diabetic groups prior to all statistical comparisons.

### Statistical analysis

The description of the biometric components was assessed using the mean, standard deviation, and 95% Confidence Intervals (CI) in two groups of participants with and without diabetes. The differences in the mean biometrics of the diabetic and non-diabetic groups were investigated using the t-test. The linear regression models were used to investigate the relationship between diabetes and biometrics while considering other independent variables. Multiple linear regression models were adjusted for the following covariates: age (continuous, years), sex (male/female), spherical equivalent (continuous, diopters), body mass index (continuous, kg/m^2^), hypertension (yes/no), and years of education (continuous). Only the right eye was analyzed to avoid within-subject dependence, a standard approach in large population-based studies. Right-left eye correlations for the primary biometric parameters were strong (range 0.76–0.94), further supporting this approach. Prior to analysis, all ocular biometric variables were examined for physiologically implausible values based on established clinical norms. For each outcome, values falling outside the following ranges were considered implausible and excluded from that specific analysis: AL: 20–30 mm (7 cases excluded); CCT: 400–650 μm (22 cases); corneal diameter (CD): 10–13 mm (19 cases); ACD: 1.5–4.5 mm (3 cases); and lens thickness (LT): 3.0–5.5 mm (65 cases). Thus, exclusions were applied on an outcome-by-outcome basis, and participants with implausible values for a given parameter were excluded only from analyses involving that parameter. All remaining values, including those beyond ± 3 standard deviations but within plausible physiological ranges, were retained. Missing data (< 2% for all variables) were handled using complete case analysis. To account for potential within-cluster correlation and its effect on standard errors and confidence intervals, all regression models were fitted using Stata’s svy command (survey methods with robust standard errors). This approach provides valid inference for cluster-sampled data without assuming independence of observations.

### Ethical approval

This study was approved by the Ethics Committee ofShahroud University of Medical Sciences, Shahroud, Iran (Referencenumber: 930/09). All procedures adhered to the tenets of the Declaration ofHelsinki. Written informed consent was obtained from all participants priorto examination.

**Table 1:** Ocular biometrics by diabetes status (Lenstar LS 900; mean ± SD). Mean differences (95% CI) and p-values. Shahroud, Iran, 2014.

Biometric components	Mean (standard deviation)			Mean difference (95% CI)	*P* value
	Non-diabetic	Diabetic			
Axial length (mm)	23.18 (0.94)	23.09 (0.98)		− 0.09 (− 0.16 to − 0.03)	0.007
Aqueous depth (mm)	2.59 (0.36)	2.56 (0.40)		− 0.03 (− 0.06 to − 0.01)	0.018*
Anterior chamber depth (mm)	3.12 (0.36)	3.09 (0.40)		− 0.03 (− 0.053 to 0.002)	0.074*
Central corneal thickness (µm)	524.25 (31.74)	530.14 (31.99)		5.90 (3.70 to 8.10)	< 0.001
Lens thickness (mm)	4.38 (0.29)	4.43 (0.32)		0.06 (0.03 to 0.08)	< 0.001*
Flat meridian (Diopter)	43.84 (1.59)	44.00 (1.54)		0.16 (0.05 to 0.27)	0.004
Flat meridian degree	86.31 (63.30)	85.41 (60.10)		− 0.90 (− 5.24 to 3.44)	0.685*
Steep meridian (Diopter)	44.68 (1.66)	44.88 (1.64)		0.20 (0.09 to 0.32)	0.001
Steep meridian degree	89.88 (44.01)	89.32 (48.29)		− 0.56 (− 3.93 to 2.81)	0.745*
Corneal Astigmatism (Diopter)	− 0.84 (0.82)	− 0.89 (0.77)		− 0.04 (− 0.10 to 0.02)	0.149
Corneal Astigmatism )degree(	86.31 (63.30)	85.41 (60.11)		− 0.90 (− 5.36 to 3.56)	0.685*
Corneal diameter (mm)	11.84 (0.44)	11.74 (0.45)		− 0.10 (− 0.13 to − 0.07)	< 0.001
Pupil diameter (mm)	4.07 (0.75)	4.04 (0.73)		− 0.03 (− 0.09 to 0.02)	0.226

*Satterthwaite approximation was used for t-tests because of unequal variances of outcome in two studied groups.

**Table 2:** Multiple linear regression coefficients and 95% confidence intervals (in parentheses) for the association between each ocular biometric parameter (rows) and independent variables (columns), including diabetes (coded as 1 = diabetic, 0 = non-diabetic), age, sex, spherical equivalent, BMI, hypertension (coded as 1 = Hypertensive, 0 = non-hypertensive), and education years. All models were adjusted for all covariates shown. Shahroud, Iran, 2014.

Biometric components	Age (Year)	Female sex	Education (year)	Hypertension	BMI (kg/m^**2**^**)**	Diabetes	SE (diopter)
Axial length (mm)	− 0.000 (− 0.006 to 0.006)	− 0.445** (− 0.508 to – 0.382)	0.016** (0.010 to 0.021)	0.027 (− 0.025 to 0.079)	0.009** (0.005 to 0.014)	− 0.111* (− 0.172 to − 0.051)	− 0.214* (− 0.336 to − 0.092)
Aqueous depth (mm)	− 0.008** (− 0.011 to − 0.006)	− 0.101** (− 0.126 to – 0.076)	0.004* (0.001 to 0.006)	0.015 (− 0.005 to 0.034)	0.005** (0.003 to 0.008)	− 0.027* (− 0.053 to − 0.001)	− 0.032** (− 0.050 to − 0.015)
Anterior chamber depth (mm)	− 0.008** (− 0.010 to − 0.006)	− 0.100** (− 0.124 to – 0.075)	0.005** (0.002 to 0.007)	0.015 (− 0.004 to 0.035)	0.005** (0.003 to 0.008)	− 0.021 (− 0.047 to 0.006)	− 0.032** (− 0.049 to − 0.014)
Central corneal thickness (µm)	− 0.035 (− 0.197 to 0.127)	1.197 (− 0.774 to 3.169)	0.717** (0.509 to 0.927)	0.108 (− 1.683 to 1.899)	0.054 (− 0.139 to 0.247)	6.751** (4.459 to 9.043)	0.900* (0.362 to 1.438)
Lens thickness (mm)	0.016** (0.015 to 0.018)	− 0.020* (− 0.039 to − 0.002)	− 0.001 (− 0.003 to 0.001)	− 0.011 (− 0.028 to 0.006)	− 0.003* (− 0.005 to − 0.001)	0.047** (0.026 to 0.068)	0.015* (0.007 to 0.024)
Corneal diameter (mm)	− 0.009** (− 0.011 to − 0.007)	− 0.115** (− 0.145 to − 0.085)	0.012** (0.009 to 0.015)	− 0.006 (− 0.033 to 0.020)	0.001 (− 0.002 to 0.003)	− 0.063** (− 0.097 to − 0.028)	0.003 (− 0.002 to 0.008)
Corneal flat meridian (Diopter)	0.018** (0.011 to 0.026)	0.697** (0.597 to 0.798)	− 0.007 (− 0.018 to 0.005)	− 0.020 (− 0.119 to 0.079)	− 0.005 (− 0.015 to 0.004)	0.076 (− 0.048 to 0.199)	− 0.034* (− 0.064 to − 0.003)
Corneal steep meridian (Diopter)	0.021** (0.012 to 0.030)	0.725** (0.620 to 0.831)	− 0.012* (− 0.024 to − 0.00)	0.005 (− 0.100 to 0.109)	− 0.003 (− 0.014 to 0.007)	0.088 (− 0.042 to 0.218)	− 0.096* (− 0.159 to − 0.033)
Corneal astigmatism (Diopter)	− 0.002 (− 0.006 – to 0.002)	− 0.028 (− 0.075 to 0.018)	0.005* (0.000 to 0.011)	− 0.025 (− 0.071 to 0.021)	− 0.002 (− 0.006 to 0.003)	− 0.013 (− 0.065 to 0.040)	0.062* (0.023 to 0.101)

**P < 0.001, *P < 0.05; SE: Spherical Equivalent by subjective refraction. Bold values indicate statistical significance for diabetes.

**Fig. 1:**
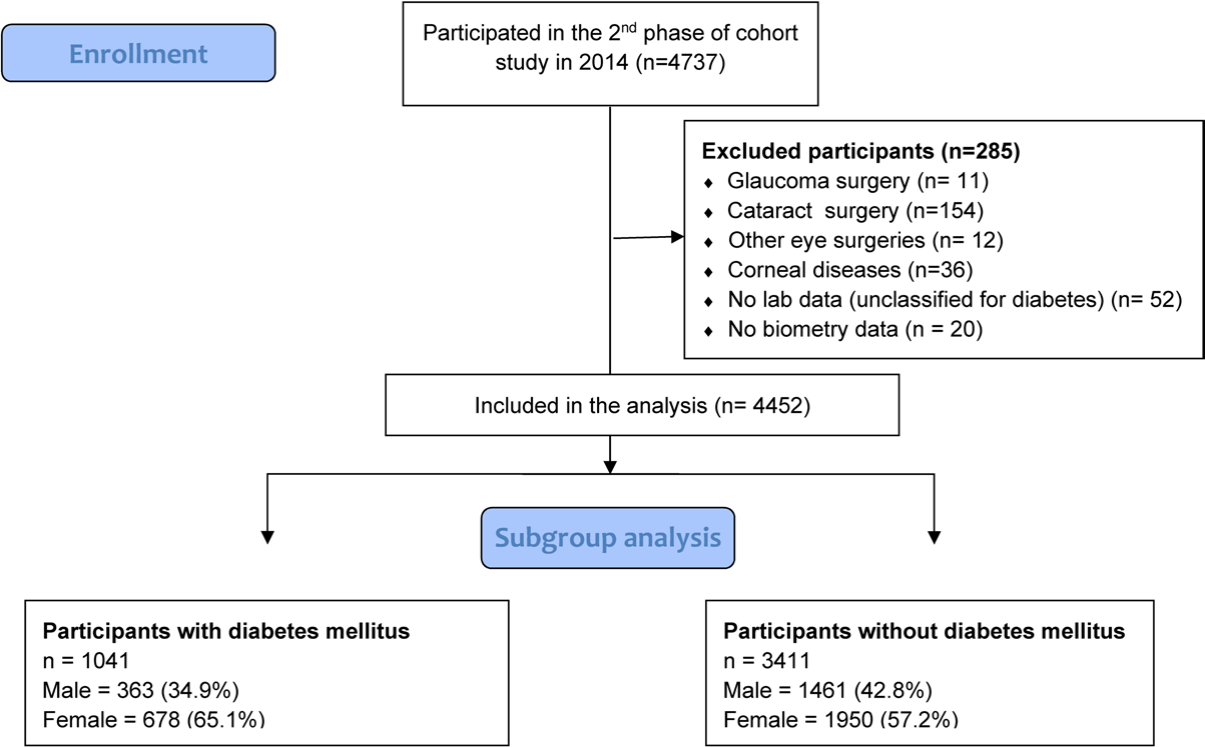
The flowdiagram of study.

## Results

Of 4737 participants in the second phase of the Shahroud Eye Cohort Study, 4452 met eligibility criteria. After applying exclusion criteria, the final analytic sample comprised 1041 participants with diabetes and 3411 without diabetes (Fig. 1). The overall mean age of participants was 55.7 years (SD 6.2; range 45–70 years), and 59.0% were women. Diabetic participants were older (mean [SD]: 57.0 [6.3] vs. 55.3 [6.1] years) and more frequently female (65.1% vs. 57.2%) than non-diabetic participants (*p* < 0.001 for both comparisons). Ocular biometric data, including group-specific means, mean differences, 95% confidence intervals, and t-test p-values, are presented in Table 1.

Unadjusted t-tests revealed significant differences between diabetic and non-diabetic participants for several ocular biometrics: AL, (*p* = 0.007), AD, (*p* = 0.018), CD, (*p* < 0.001), LT, (*p* < 0.001), and CCT, (*p* < 0.001). After adjusting for age, sex, spherical equivalent, BMI, hypertension, and education years in multiple linear regression models, diabetes remained significantly associated with shorter AL (β = − 0.11 mm, *p* = 0.007), shallower AD (β = − 0.027 mm, *p* = 0.039), smaller CD (β = − 0.063 mm, *p* < 0.001), thicker CCT (β = +6.75 μm, *p* < 0.001), and thicker LT (β = +0.05 mm, *p* < 0.001). Steep and flat corneal meridian measurements were not significantly associated with diabetes in adjusted analyses (Table 2). No other biometric parameters showed significant associations with diabetes (Table 2).

## Discussion

The present study compared the ocular biometric parameters between diabetic and non-diabetic populations and found that diabetes was associated with increased LT and CCT but decreased CD. We also found significant association between diabetes and both AL and AD such that these occular biometrics were decreased in diabetic participants.

Lens thickness was significantly greater in participants with diabetes compared to non-diabetics (mean difference: 0.06 mm; *p* < 0.001). This finding aligns with several previous reports demonstrating an association between increased LT and hyperglycemic conditions^14–16^. The underlying mechanism is well established: chronic hyperglycemia leads to intracellular sorbitol accumulation via the aldose reductase pathway, creating an osmotic gradient that drives water influx into the lens fibers, resulting in lens swelling and increased thickness^17^.

However, the literature on LT in diabetes shows considerable heterogeneity. An animal study in rabbits reported decreased LT in the hyperglycemic group, attributed to degenerative changes in lens fibers rather than osmotic swelling^18^. Another study found a slight decrease in LT following insulin treatment, suggesting that insulin-induced hypoglycemia may reduce lens water content^19^. Wiemer et al. reported no significant relationship between LT and type 2 diabetes^20^, whereas recent studies in patients with cataracts in Thailand^21^ and other studies in type 1 diabetes^22–24^ demonstrated greater LT in diabetic patients, consistent with our results.

Several factors may explain these discrepancies, including differences in diabetes type (type 1 vs. type 2), age distribution, presence of cataract, sample size, glycemic control at measurement, and statistical methods (e.g., adjustment for confounders, handling of outliers). Notably, the association became significant only after removing implausible values, underscoring the importance of rigorous data cleaning. While our large sample size enabled detection of a statistically significant 0.06 mm difference, this magnitude is smaller than the test-retest variability of most optical biometers (approximately 0.03–0.07 mm) and is unlikely to have meaningful clinical implications for anterior chamber angle assessment or intraocular lens power calculation.

Reports of corneal thickness changes in diabetes have been also controversial. Our study reported an increased CCT in the diabetic group. Several other studies have also confirmed an increased corneal thickness in diabetes^10,21,25,26^. Corneal thickness changes can be caused by either corneal endothelial damage due to diabetes-induced metabolic stress or increased thickness of epithelium due to increased epithelial permeability^27,28^. However, Módis et al. found no changes in the CCT and corneal endothelial cell density in patients with type 2 diabetes, while people with type 1 diabetes had lesser cell density and thicker CCT^29^. Also, other studies reported no association between CCT and diabetes^14,23,30^ or its severity^31^. Again type of diabetes, study sample size and agegroup, statistical methods are the main reasons for discrepancy between results of different studies.

We found a negative association between diabetes and AL and AD parameters. Some other studies also showed shorter AL in cataract patients with diabetes^21^, shallower anterior chambers in type 1 diabetes^23^, and lower median AL in individuals without type 2 diabetes^32^. Other studies found that an increase in AL would decrease the risk of development of diabetic retinopathy^8,9,11,33,34^, and inversly associated with the severity of diabetes retinopathy^32^. The reasons for this phenomenon are unknown, but potential reasons are the anatomical changes^9^, ocular biomechanics, altered retinal blood flow, reduced retinal vascular dencity, and cytokine regulations^35^.

Previous studies have reported controversial results regarding the ACD parameter. Some studies demonstrated that diabetes did not change the ACD^19,20^. For example, He et al. found no relationship between ACD and diabetic retinopathy. They claimed that the ACD had no protective effect against diabetic retinopathy. In fact, a thicker lens and deeper anterior chamber lead to stretching and making the retina and blood vessels thinner, resulting in decreased blood flow that subsequently reduces the risk of diabetic retinopathy^9^. Similar to reasons, presented above, acute decrease in glucose concentration causes decreased ACD depth in people with diabetes. Also, ACD decreases with age and diabetes duration^36,37^ and deeper ACD can have a protective effect against diabetic retinopathy^8^. Racial differences, small sample sizes, and different age ranges of participants would be the main reasons for controversial results in other studies but the most important strength in this study is the large sample size.

Multiple linear regression analysis revealed that diabetes significantly reduced AL (β = − 0.111, *p* < 0.001) and significantly increased LT (β = 0.05 mm, *p* < 0.001), but did not significantly affect ACD (β = − 0.021, *p* = 0.124). This pattern indicates that the axial shortening in diabetic eyes is primarily attributable to reduced vitreous chamber depth rather than changes in the anterior chamber alone, despite the observed lens thickening.

Several mechanisms may explain the preferential reduction in vitreous chamber depth observed in patients with diabetes. First, advanced glycation end-products (AGEs) accumulate in the diabetic vitreous at significantly higher concentrations than in age-matched non-diabetic controls^38^. This AGE crosslinking of vitreous collagen (types II and IX) promotes collagen fibril aggregation and dissociation from hyaluronan, leading to gel contraction and volume loss. Second, diabetic choroidopathy—characterized by choroidal vascular dropout and thinning—directly reduces the posterior boundary of the vitreous chamber^39,40^. Third, AGE crosslinking of posterior scleral collagen increases matrix stiffness; diabetic sclera demonstrates an 87% greater age-related increase in stiffness compared to non-diabetic controls^41^. This posterior scleral stiffening would selectively resist vitreous chamber elongation while sparing the anterior segment.

Corneal diameter was significantly smaller in diabetic participants (mean difference: − 0.10 mm; 95% CI − 0.13 to − 0.07 mm; β = − 0.063). This modest but consistent difference may reflect AGE-mediated collagen crosslinking in the corneal periphery, limiting limbal expansion. Alternatively, reduced CD may be part of a broader pattern of axial shortening (including shorter AL) observed in our findings. While a 0.10 mm difference is unlikely to have clinical significance, our finding contributes to the emerging characterization of diabetes-associated ocular structural changes beyond the posterior segment.

The large sample size (*N* = 4,452) in our study enabled detection of very small between-group differences that may not be clinically meaningful. The observed differences in CCT (+ 6.8 μm), AD (-0.03 mm), and AL (− 0.11 mm) are modest. A 6.8 μm CCT difference corresponds to approximately 0.3 mmHg of Goldmann applanation tonometry error—well within routine measurement variability. The 0.11 mm AL difference translates to 0.25–0.35 diopters of refractive prediction error, which is comparable to the test-retest variability of modern biometers and smaller than the step sizes used in IOL formulas. The 0.03 mm AD difference is below the precision of most clinical devices. Therefore, while our findings confirm statistically significant ocular biometric differences associated with diabetes, these differences are unlikely to alter clinical decision-making in individual patients. The primary contribution of this study is to characterize the very subtle structural phenotype of diabetic eyes rather than to identify clinically actionable thresholds. Although several covariates (including education, age, sex, SE, and BMI) showed significant associations with ocular biometrics in Table 2, these were not the primary focus of our study. Education may serve as a proxy for socioeconomic status or health literacy, but exploring these associations in detail is beyond the scope of this report. Nonetheless, we observed that higher education was positively associated with AL, AD, ACD, CCT, CD, and astigmatism, and negatively associated with K2 (all *p* < 0.05). These findings are exploratory and require confirmation in future studies specifically designed to examine socioeconomic determinants of ocular biometry.

Large population-based sample size in a well-executed study, standardized single-device biometry (Lenstar LS 900), rigorous diabetes classification (FBS and HbA1c), strict exclusion of prior ocular pathology, and comprehensive covariate adjustment (age, sex, spherical equivalent, BMI, hypertension, and years of education) are among the strengths of the current study. These methodological strengths position our study to provide more reliable and definitive estimates of diabetes-associated biometric changes compared to previous reports with smaller sample sizes, inconsistent measurement protocols, or less comprehensive covariate adjustment. However, Several limitations should be acknowledged. First, the cross-sectional design precludes causal inference; we cannot determine whether the observed biometric differences precede or result from diabetes. Second, although we adjusted for multiple potential confounders, residual confounding by unmeasured factors (e.g., diabetes duration, glycemic control over time) cannot be entirely excluded. Third, our findings may not be generalizable to younger populations or non-Iranian ethnic groups, as all participants were middle-aged to older adults from the Shahroud region of Iran. Fourth, corneal diameter measurement by optical biometry has lower precision than axial length measurement. Fifth, while we analyzed only the right eye of each participant to avoid within-subject dependence, this approach may not capture potential inter-eye asymmetry. Sixth, Our diabetic group included both newly diagnosed participants and those already receiving glucose-lowering medications. These subgroups may differ in terms of diabetes duration, glycemic control, and risk of ocular complications. However, exploring the effects of diabetes duration or glycemic control on ocular biometrics was beyond the scope of this study and is a topic for future research. Finally, the observed differences, while statistically significant, are small and of uncertain clinical significance.

## Conclusion

The present study demonstrates that diabetes is associated with a distinct biometric profile characterized by increased CCT and LT, and decreased CD, AL, and AD. These findings contribute to understanding the structural phenotype of the diabetic eye. However, given the small magnitude of these differences, their clinical relevance is limited. Further research, particularly longitudinal studies, is warranted to elucidate the underlying mechanisms of these biometric changes—especially the role of advanced glycation end-products and tissue biomechanics—and to determine whether similar findings emerge in other populations. Although the potential protective effect of longer axial length and deeper anterior chamber depth against diabetic retinopathy progression has been suggested in previous studies, this association was not examined in the present study, as our primary objective was to compare biometrics between diabetic and non-diabetic individuals. Future studies specifically designed to investigate the relationship between ocular biometrics and diabetic retinopathy progression are warranted.

## Data Availability

The datasets generated and/or analyzed during the current study are not publicly available due to ongoing reports that are not yet prepared, but are available from the corresponding author on reasonable request.

## Abbreviations

FBS: Fasting blood sugar
AL: Axial length
AD: Aqueous depth
ACD: Anterior chamber depth
CCT: Central corneal thickness
AL/CR: Axial length-to-corneal radius ratio
LP: Lens power
LT: Lens thickness
WTW: White-to-white distance (corneal diameter)
SD: Standard deviation
CI: Confidence interval
